# How long should we follow up patients with papillary thyroid carcinoma?: A case report describing brain metastasis after 8 years

**DOI:** 10.1097/MD.0000000000049774

**Published:** 2026-07-17

**Authors:** Hui Li, Yidong Zhu, Xiaozhen Zhao

**Affiliations:** a Department of General Practice, Xie Tu Community Health Service Center Xu Hui District, Shanghai, China; b Department of Traditional Chinese Medicine, Shanghai Tenth People’s Hospital, Tongji University School of Medicine, Shanghai, China; c Department of Medical Oncology, Longhua Hospital, Shanghai University of Traditional Chinese Medicine, Shanghai, China.

**Keywords:** brain metastasis, imaging-based surveillance, long-term follow-up, papillary thyroid carcinoma, thyroid cancer surveillance

## Abstract

**Rationale:**

Papillary thyroid carcinoma (PTC) is the most common type of thyroid cancer, characterized by favorable prognosis and low incidence of distant metastasis. However, brain metastasis from PTC is rare, and its development years after the initial diagnosis is even more uncommon. Given the potential clinical implications of late metastasis, there is a need to reconsider follow-up strategies for high-risk PTC patients.

**Patient concerns:**

A 54-year-old woman with a history of PTC diagnosed 8 years earlier presented with progressive headaches and mild left-sided hemiparesis. She had previously undergone multiple surgeries and radioiodine therapy after the initial diagnosis and had a history of childhood neck irradiation.

**Diagnoses:**

Imaging revealed a brain mass, which was confirmed as isolated brain metastasis from PTC following biopsy after surgical resection. Despite persistently undetectable serum thyroglobulin (Tg) levels and no lymph node metastasis at the time of primary surgery, this patient developed isolated brain metastasis 8 years after initial treatment.

**Interventions:**

The patient underwent craniotomy to remove the metastatic brain lesion. No adjuvant radiotherapy was given postoperatively.

**Outcomes:**

The patient recovered well after surgery, with mild left-sided hemiparesis (4/5 muscle strength). At the most recent follow-up, no additional distant metastases were detected.

**Lessons:**

This case highlights the importance of extending the follow-up period for high-risk PTC patients, including those with prior neck irradiation or aggressive tumor features. Late metastasis can occur even in patients with initially favorable prognoses and normal biochemical markers. Imaging-based surveillance is necessary to detect late metastasis early. The findings support extended follow-up strategies to enable timely intervention and improve outcomes in high-risk patients with PTC.

## 1. Introduction

Papillary thyroid carcinoma (PTC) is the most common type of thyroid cancer and accounts for approximately 80% of all cases.^[1]^ The median age of diagnosis is forty-four, and women are more frequently affected than men.^[2]^ Despite its high incidence, PTC is associated with a low mortality rate, with the majority of patients responding well to surgical resection and adjuvant radioactive iodine therapy.^[3]^ It generally has a good prognosis characterized by a relatively low incidence of distant metastasis.^[4]^ However, in some cases, PTC may exhibit aggressive behavior associated with factors such as epithelial-mesenchymal transition, epigenetic changes, or alterations in tumor cell metabolism, which can increase the likelihood of distant metastasis and postoperative recurrence.^[5,6]^ At initial diagnosis, metastases are present in about 10% of patients.^[4]^ Distant metastases in PTC most commonly involve the lungs, bones, and liver.^[7,8]^ Brain metastasis remains rare and affects only 0.1% to 5% of patients with metastatic PTC.^[9,10]^ The presence of brain metastasis presents a significant clinical challenge and highlights the need for a comprehensive understanding of the metastatic potential of PTC.

This case report discusses a 54-year-old female patient who developed isolated brain metastasis 8 years after the initial treatment of PTC. The case highlights the potential for late-stage metastasis particularly in high-risk patients with a history of radiation exposure and abnormal tumor biology. The occurrence of metastasis after such a prolonged interval raises questions about the adequacy of current follow-up protocols and suggests that extended surveillance may be necessary for high-risk patients. Unlike previously reported cases of late brain metastasis in PTC,^[2]^ our patient had persistently undetectable serum thyroglobulin (Tg) levels and no lymph node involvement at the time of initial surgery. These findings challenge the conventional approach to surveillance, which typically relies on elevated Tg levels or lymphatic spread as primary indicators for follow-up in PTC patients.^[11,12]^ This case underscores the need to reconsider follow-up strategies to better detect rare but clinically significant late complications in PTC.

Through this report, we aim to explore the clinical implications of this rare brain metastasis and provide recommendations for optimizing follow-up and management strategies for patients facing this uncommon but serious late complication.

## 2. Ethics statement

This study was approved by the Ethics Committee of Longhua Hospital, Shanghai University of Traditional Chinese Medicine (2021LCSY058). Written informed consent was obtained from the patient for publication of this case report and any accompanying images. All procedures performed in this study involving human participants were performed in accordance with the ethical standards of the institutional and/or national research committee and with the 1964 Helsinki Declaration and its later amendments or comparable ethical standards.

## 3. Case presentation

A 54-year-old Chinese female presented in June 2025 with a 1-month history of progressive headache, primarily localized to the right temporal and parietal regions. The headache was described as dull and persistent, with intermittent episodes of nausea but no vomiting or visual disturbance. Neurological examination revealed mild left-sided hemiparesis (4/5 muscle strength) but preserved cognition, speech, and coordination. The patient had a medical history of PTC diagnosed 8 years earlier and underwent 2 thyroid surgeries. She also reported childhood neck irradiation at age 8 for benign cervical lymphadenitis. She denied any family history of cancer or hereditary syndromes.

### 3.1. Initial diagnosis and management

On May 28, 2017, the patient underwent radical left thyroidectomy at a tertiary care center after presenting with a palpable neck mass. Intraoperatively, a firm, ill-defined 4.0 × 3.5 cm mass was identified in the left thyroid lobe. Extensive left cervical lymphadenopathy was observed across levels II-VI, with the largest node measuring 2.0 × 2.5 cm. A 6 × 2 cm tumor thrombus was also observed within the left internal jugular vein. Postoperative pathology confirmed classic-type PTC infiltrating fibrous tissue and skeletal muscle, with tumor dimensions of 3.0 × 2.5 × 2.5 cm. No lymph node metastases were detected in the dissected 34 nodes. Immunohistochemistry showed epidermal growth factor receptor (EGFR)^+^, p21^−^, p53^−^, 45% proliferating nuclear cell antigen (PCNA)^+^, epithelial membrane antigen (EMA)^+^, and CD15^−^. The B-Raf proto-oncogene (B-RAF) V600E mutation was not tested at that time. Following surgery, the patient received 8 cycles of radioiodine therapy. Post-treatment whole-body scans showed no evidence of distant metastasis, and serial serum Tg levels remained undetectable under suppressive therapy during initial follow-up.

### 3.2. Disease recurrence and second surgery

In late 2019, follow-up ultrasonography identified multiple calcified nodules in the residual right thyroid lobe. These nodules gradually increased in size, with the largest measuring 1.5 cm by October 2020. Given the medical history, a completion thyroidectomy was performed on October 31, 2020. Histopathological examination confirmed PTC once again, without extrathyroidal extension or vascular invasion. Immunohistochemistry showed cytokeratin (CK)19^+^, thyroid transcription factor-1 (TTF-1)^+^, Galectin 3^+^, Hector Battifora mesothelial-1 (HBME-1)^+^, calcitonin^+^, 5% Ki-67^+^, checkpoint protein (Chkpt)^+^, and thyroid peroxidase (TPO)^−^. No lymph node involvement was observed. Following the second surgery, the patient resumed levothyroxine therapy at a suppressive dose. She was monitored regularly with serum Tg levels, neck ultrasounds, and chest imaging every 6 to 12 months. Over the next 5 years, she remained asymptomatic, with no signs of local recurrence or distant metastasis.

### 3.3. Late brain metastasis

**Figure 1.**
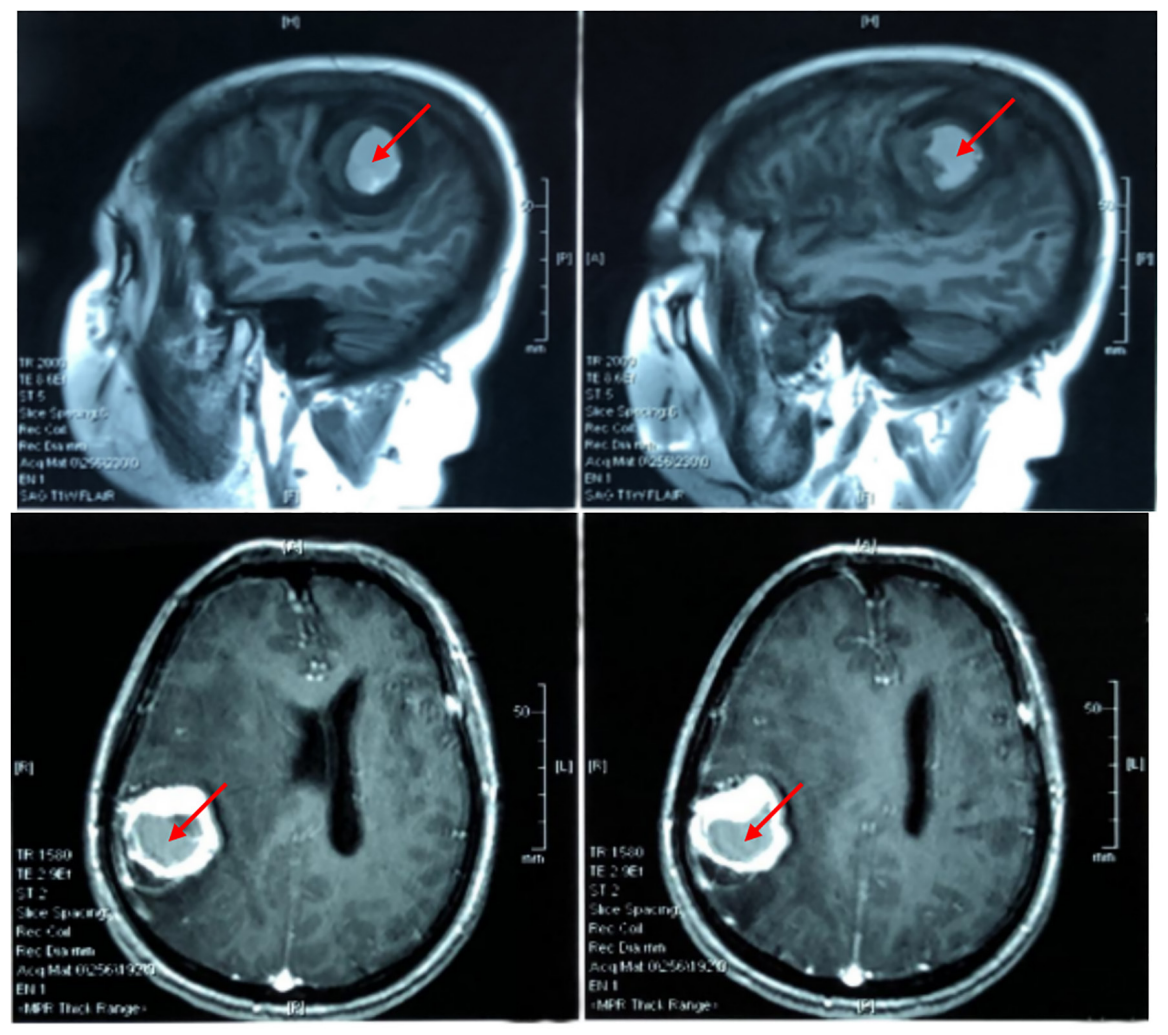
MRI scan of a solid tumoral mass at the right parietal lobe. MRI = magnetic resonance imaging.

**Figure 2.**
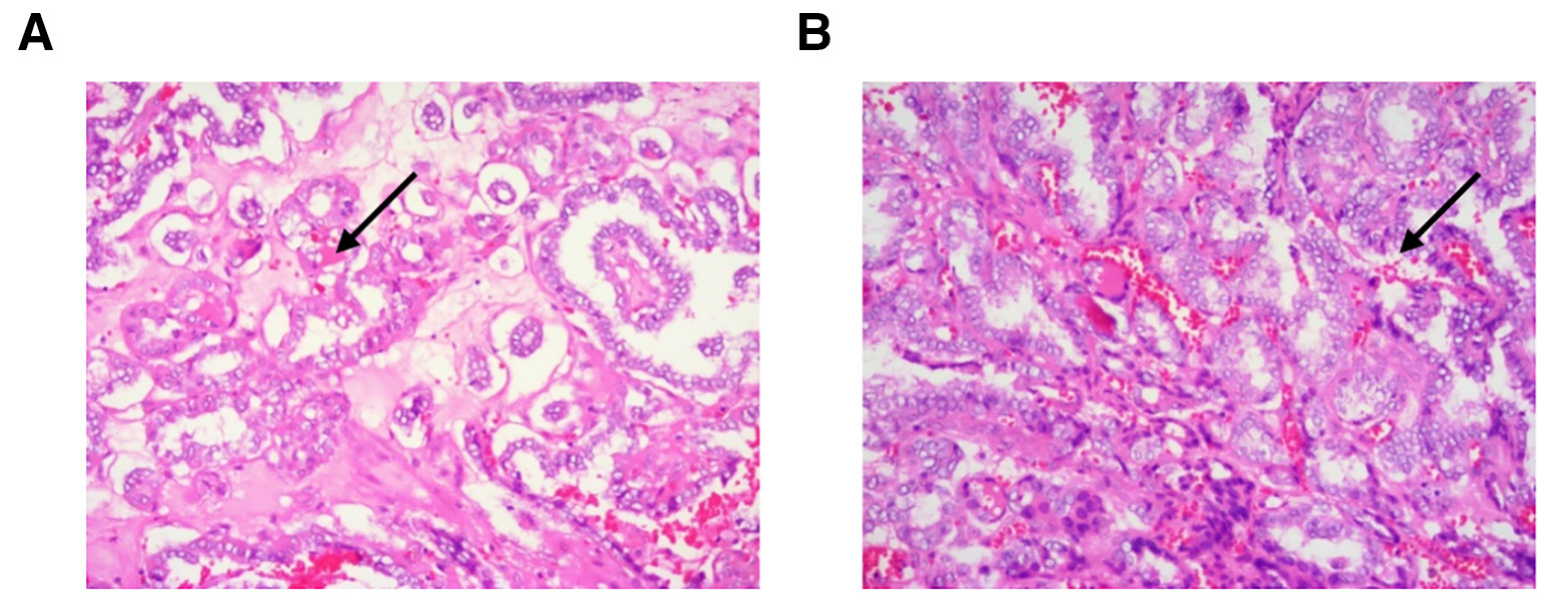
Histological examination of brain metastasis from papillary thyroid carcinoma (H&E staining). (A and B) The images showing characteristic papillary structures and nuclear features. Scale bar = 200 μm.

**Figure 3.**
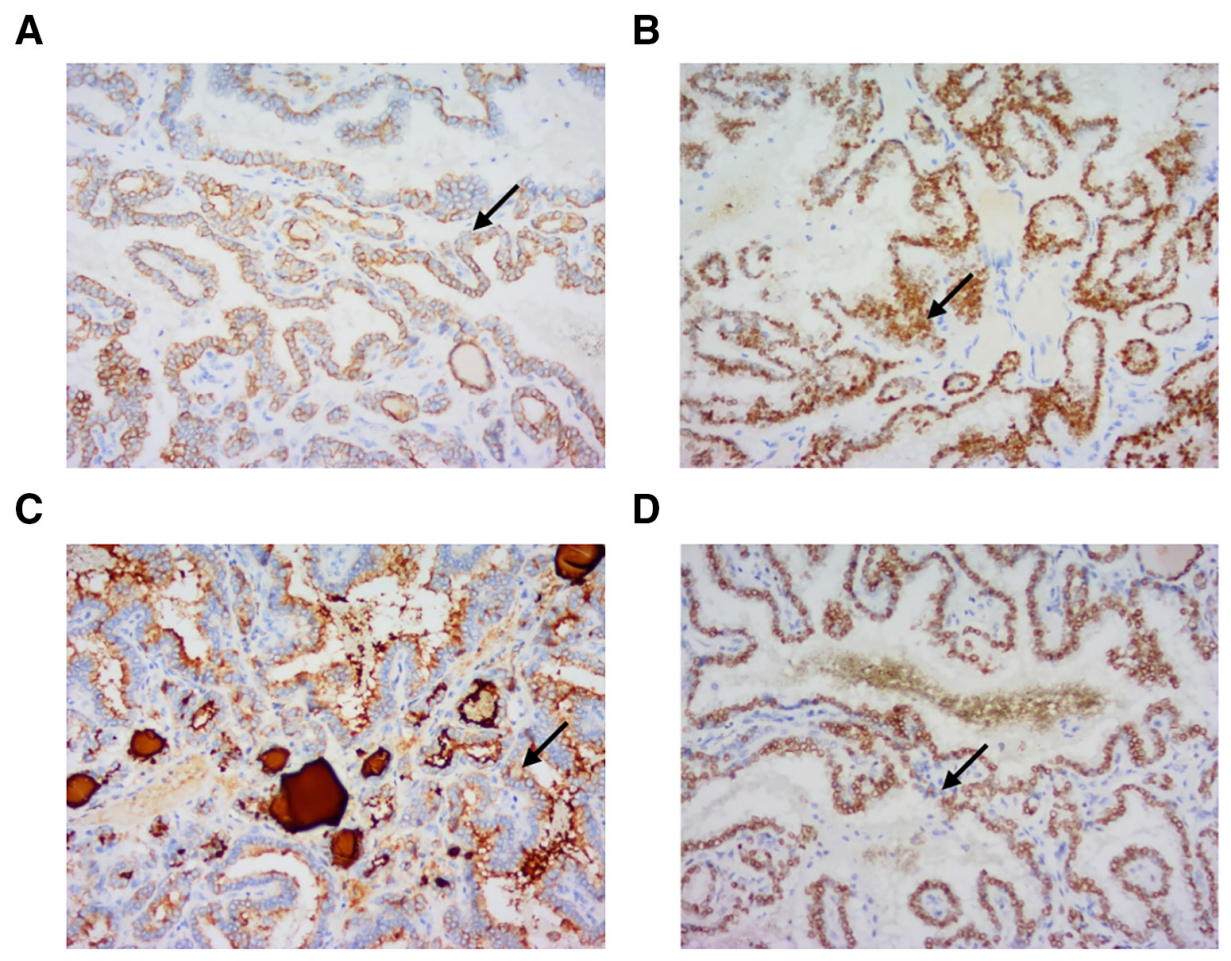
Immunohistochemical staining of the metastatic lesion. (A) CK positivity (arrows). (B) PAX8 positivity (arrows). (C) Tg positivity (arrows). (D) TTF-1 positivity (arrows). CK = cytokeratin, PAX8 = paired box gene 8, Tg = thyroglobulin, TTF-1 = thyroid transcription factor-1.

In June 2025, the patient presented with new-onset headache. Cranial magnetic resonance imaging (MRI) revealed a 4.5 × 3.8 × 3.5 cm heterogeneously enhancing mass in the right parietal lobe, with localized edema and mild midline shift (Fig. 1). No other intracranial or spinal lesions were identified. Whole-body 18F-FDG PET/CT showed no additional metastatic sites, including lungs, liver, or bones. Given her oncologic history, the lesion was suspicious for metastasis. In July 2025, the patient underwent a stereotactic craniotomy with gross total resection. Intraoperatively, the tumor showed a firm capsule and rich vascularity, which was consistent with well-differentiated metastasis. Postoperative pathology confirmed the diagnosis of metastatic PTC (Fig. 2). Immunohistochemistry showed CK^+^ (Fig. 3A), paired box 8 (PAX8)^+^ (Fig. 3B), TG^+^ (Fig. 3C), and TTF-1^+^ (Fig. 3D). Molecular testing showed no B-RAF mutation; however, telomerase reverse transcriptase (TERT) promoter analysis could not be performed due to sample limitations. The patient had a good postoperative recovery, with mild residual limb weakness (4^+^/5) but preserved cognitive and language function. Given the solitary lesion, complete resection, and patient preference, no adjuvant whole-brain radiotherapy or stereotactic radiosurgery was administered. The patient continues levothyroxine therapy at a suppressive dose and receives traditional Chinese medicine support as adjunctive care.

## 4. Discussion

PTC generally has a favorable prognosis, with distant metastasis occurring in only a small proportion of patients.^[13]^ However, several factors contribute to a poorer prognosis, including older age at diagnosis, distant metastases, extrathyroidal extension, vascular invasion, and areas of solid or poorly differentiated growth.^[14,15]^ Research indicates that approximately 70% of patients with PTC experience lymphatic spread.^[16]^ But brain metastasis is exceptionally rare. While metastasis can be associated with worsened outcomes and an increased risk of recurrence, this case introduces a unique presentation of isolated brain metastasis occurring 8 years after initial treatment. This underscores the potential for late-onset metastatic disease in PTC, even when the initial prognosis appears favorable.

Current guidelines recommend follow-up for 5 to 10 years in patients with PTC, but this case suggests that the risk of metastasis may extend beyond this period.^[17,18]^ This raises important concerns about the need for longer follow-up in high-risk patients, including those with a history of radiation exposure, vascular invasion, or aggressive tumor characteristics. The presence of childhood neck irradiation and vascular invasion with negative lymph nodes in this case is not inconsistent. Radiation exposure can induce genomic instability and promote aggressive tumor behavior, and vascular invasion enables hematogenous spread independent of lymphatic involvement.^[19]^ Therefore, negative lymph node status alone does not rule out the risk of distant metastasis when these additional risk factors are present. Based on this case and existing literature, we suggest that high-risk PTC patients require extended follow-up beyond 10 years, potentially for life.^[20,21]^ High-risk factors include: a history of head/neck irradiation, vascular invasion, primary tumor size >4 cm, extrathyroidal extension, high proliferative index (Ki-67 > 5% or PCNA positive), and/or molecular alterations such as TERT promoter mutations.^[22,23]^ The absence of lymph node metastasis should not result in downstaging for these patients. For high-risk patients, we recommend a risk-stratified surveillance approach. Brain MRI with contrast is the preferred modality due to its superior soft-tissue resolution.^[24,25]^ Given the low incidence of brain metastasis within the first 5 years, we suggest initiating screening at year 5, with MRI every 2 years until year 10, and every 3 to 5 years thereafter. This schedule balances clinical utility and cost and focuses on patients with multiple high-risk features. PET/CT should be reserved for cases with new symptoms or suspected extracranial disease. Immediate imaging is necessary if neurological symptoms, such as headaches or focal deficits, arise. For cases with uncertain biochemical markers, the abovementioned imaging techniques like MRI and PET/CT may be critical for detecting metastatic lesions early. However, we acknowledge that the proposed imaging schedule is hypothesis-generating and based on clinical reasoning: the low but persistent annual incidence of late brain metastasis, the risk of delayed diagnosis with symptom-triggered imaging alone, and practical considerations of balancing cost and patient burden. Symptom-triggered imaging remains essential for any patient developing neurological signs, regardless of scheduled intervals. Further health-economic research is needed to validate this approach.

The uniqueness of this case lies in the delayed development of brain metastasis despite persistently undetectable serum Tg and negative lymph node status. Late brain metastases from PTC have been reported previously^[26,27]^; however, those cases often involved biochemical evidence of recurrence. In contrast, our patient showed no such indicators at initial surgery. This observation suggests that a subset of high-risk PTC patients may develop brain metastasis without conventional warning signs. Therefore, imaging-based surveillance may be necessary in addition to routine serum marker monitoring. Relying solely on Tg levels or lymph node involvement may fail to identify rare but clinically significant late metastases. This case challenges the traditional follow-up approach and highlights the limitations of current guidelines that often highlight biochemical recurrence or lymphatic spread as triggers for continued surveillance.^[28]^ It is essential to reassess surveillance strategies to better detect rare but significant late complications in high-risk PTC patients, even when Tg remains undetectable and lymph nodes are negative. The urgency of imaging surveillance is further underscored by survival data for PTC patients who develop brain metastasis. Overall outcomes in this population are generally poor, with median survival ranging from 4 to 24 months across published series.^[2]^ However, patients with isolated, completely resected lesions and no extracranial disease tend to fare better, with some reports indicating median survival exceeding 3 years.^[29,30]^ Importantly, data specifically stratified by Tg status at the time of brain metastasis diagnosis are extremely scarce; most series do not report Tg levels, and those that do are biased toward patients with known biochemical recurrence. While we cannot definitively conclude whether Tg-negative brain metastases have better or worse outcomes than Tg-positive ones, the potential for irreversible neurological morbidity, such as permanent deficits from mass effect or increased surgical risk with larger lesions, justifies a proactive imaging surveillance approach in high-risk patients, regardless of biochemical status.

In terms of treatment strategy, this case shows that even after the development of brain metastasis, aggressive surgical removal can preserve neurological function and quality of life when carefully selected. Our patient underwent complete resection of a solitary brain lesion and recovered well, with no significant cognitive decline or reduction in daily functioning. For selected patients with isolated and surgically accessible brain metastases from PTC, surgery remains the main treatment option.^[31]^ In such cases, surgical treatment is associated with better neurological outcomes and longer survival.^[32,33]^ In this case, no additional radiotherapy was given after surgery, and the patient was managed with regular MRI follow-up. This approach is supported by current evidence showing that whole-brain radiotherapy does not significantly improve survival after complete removal of a single metastasis and may negatively affect cognitive function.^[34]^ Stereotactic radiosurgery is generally reserved for cases with incomplete removal, multiple lesions, or recurrence.^[35]^ More broadly, radiotherapy is typically recommended when complete removal is not achieved, when multiple brain metastases are present, or when there is spread to the lining of the brain or postoperative recurrence. Emerging treatments, including targeted therapies such as vascular endothelial growth factor receptor or B-RAF inhibitors,^[36]^ may further improve outcomes and should be evaluated in future studies.

A limitation of this case is the absence of TERT promoter mutation analysis. Evidence increasingly shows that TERT mutations are strongly linked to distant metastasis and poor outcomes in PTC.^[37,38]^ Although our patient was B-RAF-negative, a potential TERT mutation could have contributed to the late onset of brain metastasis. RET/PTC rearrangements, which are more common in patients with prior radiation exposure, are also associated with a more aggressive phenotype.^[39,40]^ Therefore, we recommend that future case reports and prospective studies involving late metastasis in PTC patients include TERT promoter testing, as it may improve risk stratification and guide decisions on extended surveillance. Based on the emerging evidence, we suggest that TERT promoter testing should be considered routinely at initial diagnosis for high-risk PTC patients, particularly those with large tumors (>4 cm), vascular invasion, extrathyroidal extension, radiation exposure, high proliferative index, or aggressive histological variants. This may refine risk stratification and guide decisions regarding the intensity and duration of follow-up. Further research should investigate the roles of TERT mutations and RET/PTC rearrangements in the development of late metastasis to improve long-term prognosis and guide more effective follow-up strategies in PTC. Moreover, as this report is based on a single case, the findings should be viewed as hypothesis-generating rather than generalizable conclusions. The unique nature of the case limits the applicability of these results to broader populations. Further research with larger sample sizes is needed to validate these insights. In addition, regarding the patient’s childhood irradiation history, the age at exposure was 8 years, which is consistent with known risk periods for radiation-induced thyroid carcinogenesis. However, the exact radiation dose could not be retrieved from historical records, as the treatment was administered over 45 years ago at a primary care facility. Clinicians should attempt to obtain both dose and age information when available, though incomplete records are a common limitation in such cases.

In summary, this case suggests the latent risk of brain metastasis in PTC and underscores the need for extended follow-up strategies. Brain metastasis can occur in the absence of elevated serum Tg levels or lymph node involvement, suggesting that imaging-based surveillance is essential for high-risk patients. Current follow-up protocols may need to be revised to improve early detection of late-stage metastasis and ensure timely intervention. Future research should aim to identify pathological features and biological markers that predict the risk of brain metastasis and develop optimized treatment strategies for managing late-stage disease.

## 5. Conclusion

This case report suggests the potential for delayed brain metastasis in PTC even in the absence of elevated serum Tg levels or lymph node involvement. Regular whole-body and brain imaging could aid in the early detection of metastasis. Current follow-up protocols may need to be revised to include extended surveillance, improve early detection of late-stage metastasis, and facilitate timely intervention. By highlighting the clinicopathological features of this case, we aim to provide insights that may improve diagnosis, guide treatment decisions, and inform prognosis. These findings emphasize the need for extended follow-up periods and the incorporation of imaging techniques to improve survival outcomes in PTC.

## Author contributions

**Conceptualization:** Hui Li.

**Data curation:** Yidong Zhu.

**Supervision:** Xiaozhen Zhao.

**Validation:** Xiaozhen Zhao.
